# Addressing barriers in FAIR data practices for biomedical data

**DOI:** 10.1038/s41597-023-01969-8

**Published:** 2023-02-23

**Authors:** Laura D. Hughes, Ginger Tsueng, Jack DiGiovanna, Thomas D. Horvath, Luke V. Rasmussen, Tor C. Savidge, Thomas Stoeger, Serdar Turkarslan, Qinglong Wu, Chunlei Wu, Andrew I. Su, Lars Pache, the NIAID Systems Biology Data Dissemination Working Group

**Affiliations:** 1 Department of Integrative, Structural and Computational Biology, The Scripps Research Institute, La Jolla, CA 92037 USA; 2 Velsera, 529 Main St, Suite 6610, Charlestown, MA 02129 USA; 3 Department of Pathology & Immunology, Baylor College of Medicine, Houston, TX 77030 USA; 4 Texas Children’s Microbiome Center, Department of Pathology, Texas Children’s Hospital, Houston, TX 77030 USA; 5 Department of Preventive Medicine, Northwestern University Feinberg School of Medicine, Chicago, IL 60611 USA; 6 Texas Children’s Microbiome Center, Texas Children’s Hospital, Houston, TX 77030 USA; 7 Department of Chemical and Biological Engineering, McCormick School of Engineering, Evanston, IL 60208 USA; 8 Institute for Systems Biology, Seattle, WA 98109 USA; 9 Scripps Research Translational Institute, La Jolla, CA 92037 USA; 10 Department of Molecular Medicine, The Scripps Research Institute, La Jolla, CA 92037 USA; 11 Infectious and Inflammatory Disease Center, Immunity and Pathogenesis Program, Sanford Burnham Prebys Medical Discovery Institute, La Jolla, CA 92037 USA

**Keywords:** Data publication and archiving, Standards

**FAIR data sharing is integral to spur research reproducibility, promote data reuse, and accelerate research. However, the first step in using these data assets – discovering that they exist – is compounded by problems in incentives, standardization, and coordination of efforts**. In 2023, the National Institutes of Health (NIH) implemented their updated Data Sharing Policy, which mandates timely data sharing of all NIH-funded data. However, for the policy to shift the data sharing culture, to improve research reproducibility, and to promote data reuse, several changes must happen. In a companion piece^1^, we present a survey of the data sharing landscape for immune-mediated and infectious disease data, combined with our efforts to create a reusable methodology to catalog data we generate. We found that researchers routinely share data, but datasets are still not necessarily findable, accessible, interoperable, or reusable (FAIR)^2^. In the course of these efforts, we identified three interdependent barriers that need to be addressed to maximize the impact of data sharing efforts that are becoming increasingly commonplace: (*1*) a lack of incentives, (*2*) little standardization in metadata collection and access, and (*3*) uncoordinated efforts. Here, we propose solutions to improve the FAIRness of metadata, data, and the experimental conditions used to generate these data. Our solutions include leveraging journals as a key driver of research incentives and consolidating data searching and citation into a centralized platform. While we focus our discussion based on our experience primarily with NIH-funded research, these principles are broadly applicable to other data sharing efforts worldwide.

**Barrier 1: Without incentives, researchers tend to provide incomplete metadata, which limits data discovery and reuse**. Currently, there are few tangible incentives for researchers to share data. There is limited recognition amongst scientists regarding the benefits of data sharing, while the potential for downside effects such as the risk of getting scooped, concerns over protecting participant/patient privacy, a lack of Intellectual Property protection, and the enhanced workload associated with providing good, reusable, and discoverable data are all well recognized. As a result, researchers frequently do the bare minimum to comply with journal and funder mandates. When sharing data, researchers also include metadata which describe the contents of the dataset to aid in searchability, but these metadata are often insufficient to help researchers identify useful datasets. Even if a metadata property is required, researchers can circumvent these requirements by providing short, generic descriptions. Consequently, metadata is often cursory and incomplete, and data is often provided in non-machine readable formats (*e.g*., .pdf, Microsoft Word, or free text) without associated documentation to support reuse. While there are many projects that attempt to improve the FAIRness of poorly collected metadata^3,4^, it is far easier to collect complete, well-structured metadata from the start (*see Solution 2.3*). Our proposed solutions tap into the two primary incentive structures for researchers: funding opportunities and recognition via citations.

**Solution 1.1: Ensure that there are sufficient resources for data sharing activities within funding opportunities**. Documenting data to make it reusable takes time and effort, detracting from experimentation, data analysis, and synthesis of insights into publications. If funding agencies (*e.g.*, NIH) mandate data sharing, they should be prepared to increase budgets accordingly to dedicate a portion of the effort to ensuring the resulting data is findable and reusable. The new NIH policy does allow for investigators to budget for data management and data sharing (https://grants.nih.gov/grants/guide/notice-files/NOT-OD-21-015.html) but does not include dedicated funding specifically earmarked for data management and sharing, which may require an increase in budget caps to accommodate these mandated activities. Dedicated funding could support the addition of FAIR data experts within an institutional core or research teams to shepherd them through data and metadata management and curation, as well as supporting training for existing staff (*Solution 3.3*).

**Solution 1.2: Reward data sharing compliance to incentivize researchers to share data well**. In our experience, we have seen limited to no enforcement of data sharing plans, funding agency policies, or journal policies^5^, which reinforces the lack of incentives for providing discoverable data. Tracking data sharing compliance, evaluating data sharing quality, and rewarding good data sharing practices by funders may close the gaps in promoting discoverability. As metadata becomes more findable through efforts to catalog datasets and track their impact via a dataset reuse index (*see Solution 3.1*), it will be easier for funding agencies to track compliance. Evaluation criteria for funding opportunities should consider past performance in data sharing, rewarding researchers who do it well, and pushing the field towards providing timely and well-documented metadata and data. Prizes such as the DataWorks! Challenge (https://www.herox.com/dataworks/guidelines) to reward exemplars of data sharing and reuse are complementary mechanisms to incentivize good data sharing. While monetary incentives are useful to drive attention to the area, however, we believe the larger incentive lies in recognizing good data sharing within the grant review process and as a component of institutional promotions.

**Solution 1.3: Ensure all recommended data repositories provide permanent, citable identifiers**. Data reuse, a metric of researchers’ impact on the community, is one of the primary motivations for researchers to share data. To create a citable object for datasets, many repositories generate a Digital Object Identifier (DOI) for each dataset. However, DOI-generation is not free, so this practice has not been adopted by all data providers, including many recommended by the NIH (https://www.nlm.nih.gov/NIHbmic/nih_data_sharing_repositories.html). This permanent, citable identifier^6^ could be provided by a centralized organization (*see Solution 3.1*), similar to a PubMed Identifier. Moreover, the research community needs to change its culture from only citing a publication associated with a dataset to citing both the paper and the dataset. In our experience, researchers tend to cite publications even if a dataset DOI exists; as a result, the influence of the dataset is difficult to ascertain, and the location of the data is obfuscated for a researcher intending to reuse it. This type of culture change tends to be driven by the ease to which researchers can cite datasets and track reuse, and the change becomes catalytic as researchers can quantify how these metrics expand the impact of their research. We do not envision dataset citations to come at the expense of publication citations or traffic to journal websites; rather, both publications and datasets should be cited, providing exposure both to the journals and to data repositories. These changes have the added benefit that it will become easier to disambiguate publications which generate data (primary data generation) from those which reuse data (secondary meta-analyses), in addition to quantifying reuse via metrics like h-index for publications (*see Solution 3.1*).

**Solution 1.4: Leverage the critical role that publications play in researcher incentives to encourage data sharing best practices and monitor data sharing and reuse**. In our user research, data discovery often starts with a researcher reading or searching for an article on a particular topic, and then searching for the data associated with the publication. Publications therefore often represent the entry point to data discovery, and additionally provide legitimacy to a dataset by connecting the raw data with methodological descriptions, protocols, analysis, and interpretation. The recent increase in scientific preprints (e.g., bioRxiv and medRxiv) additionally highlights the need for proper data sharing to occur early in the process, at latest by the time of submission or when a research project is posted on a public-facing website. Emphasizing the growing importance of preprints within scientific research, PubMed has started to index preprints alongside peer-reviewed publications (https://www.ncbi.nlm.nih.gov/pmc/about/nihpreprints/).

Despite the interconnection between publications and data, however, dataset metadata does not always contain explicit linkages to their resulting publications, and preprints or peer-reviewed publications do not always list all their underlying datasets in structured, searchable fields. To address this gap, preprints and publications should be better connected within NIH-based data discovery projects in the United States, such as the Generalist Repository Ecosystem Initiative (GREI, https://datascience.nih.gov/news/nih-office-of-data-science-strategy-announces-new-initiative-to-improve-data-access), the Common Fund Data Ecosystem Portal (https://app.nih-cfde.org/), the National Institute for Allergy and Infectious Disease (NIAID) Data Ecosystem, and others. Journals offer a pathway forward to compliance to create these linkages: already, policies like Springer Nature’s “Data availability statements^7^” offer the first step towards ensuring data discovery associated with a given publication. However, these statements are only available in free text without explicit links to URLs, limiting their searchability. Additionally, these statements often merely state that “data is available upon request”, creating delays in accessing data, and a recent study showed that 90% of corresponding authors declined or did not respond to data requests^8^. Lastly, a large portion of “available data” is locked in Supplementary Materials, often in non-machine-readable formats, rather than in a repository with structured metadata and a persistent identifier to cite the dataset. By encouraging or mandating more structured data availability statements and dataset metadata with links to unique identifiers in a repository, preprints and journals have the opportunity to nudge researchers towards a more searchable description of the data. Sharing data alongside a publication should ideally first occur within a preprint: not only does this allow other researchers to validate the claims within the article, but in our experience, we have also benefited from community members identifying critical issues or offering suggestions on our data or analyses prior to peer review submission. Sharing a dataset linked to a preprint also provides an opportunity for researchers to begin citing the data. Journals and preprint servers also benefit from this relationship: by making datasets easy to find and use, they lower barriers for researchers to reuse the data and cite the publications associated with the data, increasing traffic to the journal and its overall reputation.

**Barrier 2: Methods to generate, combine, and access dataset metadata are unstandardized**. Metadata that completely describe the contents of a dataset and experimental conditions used to generate the data are essential to promote reuse. To define which properties need to be collected about a dataset, a schema defines the set of field names within the data and what they represent (*i.e*., description (dataset description), creator (author(s) who generated and/or processed the data), measurementTechnique (experimental technique(s) used to collect the data), etc.^1^). More detailed schemas also define the allowable values (controlled vocabularies, or ontologies, which are formal representations of allowed values and their relationship to each other) and constraints such as type or expected number for each property. Data templates or forms can then use these schemas to capture metadata in a structured and consistent manner.

However, the data sharing field is littered with the corpses of dead, abandoned, and unstandardized schemas. A large number of schemas to describe dataset metadata have been generated – and abandoned – over the years, begging the question: why have so many schemas been created? We hypothesize that this proliferation of standards arises from a combination of a lack of awareness of other standards, difficulty implementing the schemas, and the schemas being ill-suited to a particular context. As a result of these differing metadata standards, projects which attempt to aggregate dataset metadata together become challenging to maintain, curtailing the findability of datasets. We propose creating a core set of standardized properties, built off existing community standards such as Schema.org, while allowing flexibility to add additional properties for a particular use case. Tools to support the generation of standardized metadata need to be augmented, and methods to access metadata and experimental data need to be standardized.

**Solution 2.1: Endorse common but extendable metadata schemas maintained by a centralized community source**. Instead of reusing existing schemas, repositories often develop their own schemas in a vacuum, creating challenges in combining schemas from multiple sources. Often, many of these properties are semantically equivalent (for example, author and creator), suggesting that standardization across a core set of properties will be possible. Determining which fields are essential to promote discoverability is an unresolved question, and will differ based on subject area and intended reuse of the data. The NIH-funded GREI (https://datascience.nih.gov/news/nih-office-of-data-science-strategy-announces-new-initiative-to-improve-data-access), which seeks to standardize metadata across seven generalist repositories, is an encouraging start to this problem. However, in our experience, generalist schemas often lack key properties to promote reuse in biological contexts, including funding source, health condition/disease, access restrictions, collection and processing details, and links to supporting documents such as protocols and publications. Further, many institutes within the NIH endorse their own set of schemas; we need to identify commonalities between standards, endorse a core set of properties, and allow for extensions which build off this foundation (*see Solution 3.2*). Education and training will help researchers incorporate these schemas into their data management plans (DMPs) and lab operations (*see Solution 3.3*).

In a companion piece^1^, we outline a minimalistic but extendable schema, developed for capturing infectious disease Dataset and ComputationalTool metadata (NIAID SysBio schemas). Cognizant that it is highly unlikely that there will be a single schema “to rule them all”, our proposal is built off the widely used Schema.org standard with the addition of properties to promote findability of infectious disease resources, and it contains a crosswalk to 15 commonly used standards to promote interoperability between different schemas. There is a constant tension between developing broad, generalizable schemas to promote interoperability between sources and hyperspecific, domain-optimized schemas. Our approach was to establish a base of common, interoperable minimalistic dataset schemas and then add customized additions for specific use cases or thematic areas. These schemas are intentionally parsimonious to capture a key set of defining properties for biological datasets without being exhaustive, and can be extended using tools like the Data Discovery Engine^9^ to create customized schemas which inherit properties of the parent schema.

If a common schema is adopted, standardization of metadata will improve; however, encouraging community adoption of schemas is also non-trivial. ELIXIR, a European intergovernmental agency, has been implementing European FAIR data policy commitments by supporting the development and adoption of Bioschemas, along with related efforts like the FAIR Cookbook to make data FAIR. Bioschemas working groups consist of life sciences communities of practice, researchers, repositories and more, work together to develop new biologically relevant schemas, and provide training and encourage adoption of those schemas with the support of ELIXIR. Funding agencies like the NIH are in a unique position to endorse and promote a core set of properties as being essential for Dataset and ComputationalTools, while allowing for extensions to particular biological contexts or repository use cases. This endorsement would also assuage concerns about whether a schema will exist in a year, which may prevent users from adopting that schema. This work could build on the work of the Center for Expanded Data Annotation and Retrieval (CEDAR), a NIH-supported project to develop, evaluate, use, and refine biomedical metadata and metadata templates^10^. CEDAR hosted a research collection of metadata, built metadata authoring tools, and provided training for researchers to use metadata; as such, it provides an important example of an NIH-endorsed project on metadata collection and training on the use of metadata. Additionally, the creation of an organization to oversee the development, evolution, integration, and deprecation of schemas, similar to how the National Library of Medicine (NLM) terminologies connect outdated terminologies using the Unified Medical Language System (UMLS) (*see Solution 3.1*), would help establish and maintain greater standardization and interoperability.

**Solution 2.2: Provide consistent methods to preview and access metadata and data within repositories**. Often there are a number of hurdles, including institutional approvals, data usage agreements, and data access applications, before a researcher can view the contents of a dataset. In the worst cases, a researcher is forced to develop a research plan justifying access to the dataset based on hypotheses about what might be included in the data. This friction makes evaluating a dataset’s potential utility a frustrating and time-consuming process, and many researchers who could increase a dataset’s value via reuse give up rather than trying to navigate these hurdles. Data repositories should clearly display sufficient information to streamline this evaluation, including previews of a subset of the data and a description of all fields within a dataset (data dictionary), before a researcher goes through the more involved process of signing a data usage agreement or applying for access.

Once a researcher has identified that a dataset is useful, accessing its contents becomes challenging as each repository has its own way of accessing dataset metadata and data. Some have Application Programming Interfaces (APIs), but they require different inputs and produce different outputs for each repository. That’s the best case; more commonly, metadata is standardized, but inaccessible unless a developer writes a program to crawl the HTML code, resulting in slow and brittle processes and untidy metadata results. Data, especially those stored within publications, are most commonly only accessible through individual serial downloads. For metadata, the Open Archives Initiative Protocol for Metadata Harvesting (OAI-PMH)^11,12^ is a standardized API-based mechanism to access metadata which has been adopted by generalist repositories such as Figshare, Harvard Dataverse, Mendeley, and Zenodo; however, the implementations vary by source and the resulting metadata formats differ, making it impossible to write a single piece of code to access multiple repositories. Similarly, GA4GH Data Repository Service (DRS, https://ga4gh.github.io/data-repository-service-schemas/preview/release/drs-1.2.0/docs/) API combined with a passport minted by NIH Researcher Auth Service (RAS) has been adopted as a common mechanism to programmatically access data, but its adoption across biological repositories has not yet been consistent and DRS provides extremely limited metadata today. As a result, dataset aggregation projects have to write a multitude of couplers, unique to each repository, to access information. Journals do not provide programmatic ways to access data associated with publications, resulting in a swath of lost data that is “open” but not discoverable. To circumvent these issues, data repositories should coalesce on standard mechanisms to access both metadata and data. The Generalist Repository Ecosystem Initiative (GREI), which is standardizing metadata across generalist repositories, could pilot these standardization efforts and then expand them to domain-specific repositories. Providing a uniform mechanism to access metadata and data creates opportunities for platforms to easily aggregate data, provide previews of datasets to researchers before applying for access to datasets, and pipe data into cloud-based analytical workspaces.

**Solution 2.3: Continue to improve tools that streamline metadata/data collection and sharing**. Collecting and registering metadata according to a standardized schema is an onerous process, creating friction for users to make their datasets findable. To address these challenges, the Data Discovery Engine (DDE) is a project to promote FAIR data sharing practices^9^. The DDE allows researchers to find existing schemas, extend or modify existing schemas, register custom schemas, and register datasets according to a schema. As such, the DDE lowers the barrier for researchers to reuse pre-existing schemas. The DDE Metadata Registry also provides an easy-to-use interface to register dataset metadata which comply with a standard, either via a form or a file-based bulk upload system; this interface also validates submissions and links categorical properties to ontologies where possible. In a complementary effort, CEDAR has built a repository to specifically identify metadata patterns to guide predictive data entry into metadata templates^10^.

Moving forward, more tools are needed which merge human and automated curation to offload the burden of metadata creation from data creators. As the new NIH policy and other sharing mandates are implemented, the ability of different research groups will vary based on expertise and bandwidth; easy-to-use tools help level the playing field by helping researchers share metadata and data with minimal effort. Ideally, metadata submission would pull information from other sources (*i.e*., NIH RePORTER) where possible in order to provide details about funding mechanisms or other metadata and avoid duplicative entry. Tools such as the DDE Metadata Registry and CEDAR to collect robust and standardized metadata should be integrated within data repositories so users could generate metadata, upload data, and create DOIs within a single platform. Additionally, a publically-supported centralized searching mechanism (*see Solution 3.1*) would enable funding agencies to freely lookup information in real-time, rather than having to send time-consuming requests for information from grantees. By lowering the barriers to collecting complete metadata and sharing data, data FAIRness should increase while decreasing researcher effort.

**Barrier 3: Data dissemination efforts are uncoordinated, resulting in duplicative efforts and the proliferation of many redundant solutions**. In the past decade, there have been a number of efforts to improve data discovery of open data assets, focusing on developing exhaustive but difficult-to-implement schemas, aggregating research outputs such as datasets, or applying Natural Language Processing (NLP) algorithms to improve the searchability of metadata. Diverse solutions to challenges in dataset findability encourage testing new approaches and providing bespoke solutions to particular research contexts, but come at the expense of coordination and persistence. Efforts across NIH and the broader international community are often uncoordinated, leading to duplicative work and the proliferation of myriad standards and platforms. Additionally, the lack of long-term investment and the short-term funding of many of these projects means that the longevity of many of them is uncertain, leading to abandoned projects that are no longer maintained.

**Solution 3.1: Develop a centralized mechanism to search and cite datasets**. A number of projects have attempted to aggregate datasets, often for a particular research domain. While these projects fulfill a key gap – providing a unified platform to search for open biological datasets and potentially track data citations – they lack the legitimacy of a centralized solution, and the long-term outlook of many of these projects is uncertain. For instance, DataMed, a biomedical research dataset index funded under the Big Data to Knowledge Initiative, was launched in 2016 to improve discoverability of biomedical datasets^13,14^ and mapped metadata to core Data Tag Suite (DATS) elements, conducted user experience studies, and applied NLP algorithms to improve the searchability of the indexed datasets. However, in the intervening years, the DataMed site is still a beta version, the indexed datasets have not been updated since 2017, and it is unclear whether the effort will continue. Additionally, other domain-specific projects have sprung up alongside this more general solution, including efforts funded by individual NIH Institutes containing similar functionality but targeted to different research domains. As some of the builders and users of these systems, we worry that these efforts are essentially building another layer of “megarepositories” which are not interoperable or connected, on top of isolated, unstandardized repositories – not to mention potentially duplicating efforts. Given the numerous attempts by multiple groups for improving the findability of data, a unified solution backed by a respected institution offers many advantages. These include project legitimacy (promoting uptake), metadata centralization (increasing findability), de-duplication of efforts (maximizing investments), and long-term maintenance, sustainability, and preservation (ensuring projects have a sufficient timeline to realize their impact).

A centralized platform to search and cite datasets would have three key features. First, it would be a federated model that builds on top of an ecosystem of established repositories. The centralized metadata platform would store only metadata descriptions of these datasets, with the data storage distributed across existing repositories. Second, the platform should create a standard for a core set of dataset metadata (*see Solution 2.1*). Similarly to how MEDLINE has created a standard for publications to be included in PubMed, this metadata standard would define what information diverse repositories need to collect in order to be included in the searchable index, promoting interoperability. Third, each dataset should contain a unique, citable identifier, allowing tracking of dataset reuse. This tracking would enable measurable outcomes for secondary analyses of data, similar to or incorporated into the h-index, a key incentive for researchers (*see Solution 1.3*). PubMed, developed by the NLM, offers a model for how to create a centralized and standardized home for datasets assembled from repositories. PubMed is often considered the gold-standard for scientific literature queries, aggregation, curation, and citation; however, the development and integration of the processes involved in advancing PubMed to a gold standard level happened through years of iterative changes, and the inclusion of datasets could be a natural future step. NLM could be a natural partner to expand their platform to provide the equivalent of “PubMed for Datasets”. In addition to providing a unified search interface and consistent citations, researchers could also use this platform to create a curated list of their datasets, the dataset equivalent to NCBI’s MyBibliography used in NIH Biosketches. By partnering with NLM, this catalog could also create linkages between datasets and publications in PubMed, building off the addition of fields in MEDLINE in 2015 to connect datasets and publications. These linkages would make it easier for researchers to find datasets from publications and to discover and cite associated publications from a dataset, amplifying the impact of the papers. By centralizing this effort, the community would have a long-term, comprehensive solution to dataset discovery instead of the piecemeal approach that has happened thus far. Data sharing for discovery is a classic example of combining individual efforts for the public good; as a result, the most logical approach is a coordinated effort across the federal government.

**Solution 3.2: Explore how long-term funding mechanisms can sustain data projects and tools**. Often, data standards, tools, software, platforms, and resources are developed as pilot projects or as side effects of hypothesis-driven scientific grants. For example, the NIAID Systems Biology Data Dissemination Working Group developed and implemented an infectious disease-specific Dataset and ComputationalTool schema, increasing the FAIRness of nearly 400 datasets and computational tools using it^1^. The schema is straightforward, yet has potential to exponentially enhance biological and biomedical dataset accessibility and reuse via increased exposure through dataset aggregation projects like Google Dataset Search. However, this project was completed as a voluntary Working Group which included participants across the NIAID Systems Biology Centers. As a result, the long-term stability to maintain and expand such services remains in jeopardy. This instability in project longevity makes people unwilling to adopt new projects and create linkages between existing and new platforms, and often new initiatives recreate existing functionality in a defunct project whose funding expired. Recently, institutes like the National Cancer Institute (NCI)^15^, NIAID, the National Heart, Lung, and Blood Institute (NHLBI), and the National Eye Institute (NEI) have developed dedicated mechanisms to support informatics and data science research and tool development. These programs are essential to ensure investments in FAIR sharing infrastructure are not squandered by promoting sustainable research software development, fostering a robust developer community, and developing career paths for research software engineers. Even if initial funding is made explicitly for data projects and tools, dedicated, long-term funding mechanisms are needed for sustainability. For example, the BD2K program supported a number of valuable data projects including DataMed and CEDAR, but since BD2K funding ended, the future of these important endeavors remains uncertain.

**Solution 3.3: Provide training and guidance to adapt policies within the context of how a lab operates**. Even for researchers keen to disseminate their data, it is overwhelming and time-consuming to figure out where to start. It’s easy for people to agree they want to make their data FAIR; it’s harder to figure out what exactly is meant by “FAIR” or how to make data FAIR. The barrier to entry is large; for instance, when we wanted to develop the NIAID SyBio schemas, we could find no model or guidance on how to navigate this process, and instead implemented our own solution with little guidance to the best practices. Researchers are not educated on how to do data management properly from the start of the project, resulting in wasted effort at the end of a project to retrofit previously collected data. Researchers are not often aware of the best practices with data sharing, and even if they are, there are few guides for how to implement them in a biological context. Easy-to-implement, practical guidance, accompanied by training sessions, will be critical to ensure that researchers and staff take advantage of standards (*Solution 2.1*) and tools (*Solution 2.3*) that are developed to improve the sharing, discovery, and reuse of data. The NIH has begun to develop such guidance in response to their data sharing policy (https://sharing.nih.gov/data-management-and-sharing-policy/about-data-management-and-sharing-policy/data-management-and-sharing-policy-overview); such education, training, and guidance is essential to ensure the success of this policy. As a complement to this guidance, we also recommend integrating data and metadata curation and management as a core component of graduate training, supported by postdoctoral fellowship opportunities to research these topics.

## Conclusion

In recent decades, the research community has increasingly embraced data sharing to make their research accessible – a core component of making data findable, accessible, interoperable, and reusable (FAIR). The ongoing COVID-19 pandemic highlights both the scope and importance of open data sharing: an unprecedented amount of data has been generated and shared, often in real-time, which has helped the research community understand the novel virus and disease, develop effective therapeutics and vaccines, and refine public health measures to mitigate its effects. While this enthusiasm has great potential to foster reproducible research, encourage data reuse, and accelerate research, merely sharing data does not ensure that the data are FAIR.

We have identified three key barriers which have limited the impact of widespread data sharing. First, incentives to encourage researchers to share well-described and well-documented data are limited, relying instead on broad data sharing policies and/or researcher altruism. With the updated NIH Data Sharing policy that went into effect in January 2023, the biological research community is at a unique inflection point to tackle impediments in FAIR data practices, and potentially dramatically shift in the sharing of research data to reproduce research, promote data reuse, and spur research advances.

To address incentive barriers, we propose providing set-aside funding for data management efforts, rewarding good data practices, tracking reuse via citable identifiers, and leveraging the unique role of journals to encourage these efforts. Second, methods to create and access metadata are unstandardized. We suggest the creation of a core schema which encompasses a common set of properties to describe datasets, consistent methods to preview and access metadata and data, and tools which make it easy for research groups to collect and share metadata. Lastly, data discovery projects are often uncoordinated and unsustained. To address these challenges, we recommend the creation of a centralized platform to discover and cite datasets, sustained investments in data tools, and practical training that makes it easier for researchers to follow FAIR sharing best practices. All of these efforts will require a broad coalition of partners drawn across the research community, including researchers, scientific societies, repositories, funding agencies, and publishers. Our suggestions here primarily focus on the first stage of sharing, discovering, and reusing data: discovering that a dataset exists and that it might be useful. Additional challenges regarding data quality, interoperability, and curation processes are essential to understand what limits data reuse, but these efforts are meaningless without the initial step of easily finding datasets.

## Data Availability

Data sharing is not applicable to this article as no datasets were generated or analyzed during the current study.

## Code availability

Code sharing is not applicable to this article as no datasets were generated or analyzed during the current study.
